# Tobacco Smoke and CYP1A2 Activity in a US Population with Normal Liver Enzyme Levels

**DOI:** 10.3390/ijerph18052225

**Published:** 2021-02-24

**Authors:** Alexis Garduno, Tianying Wu

**Affiliations:** 1 Herbert Wertheim School of Public Health and Human Longevity Science, University of California, San Diego, CA 92093, USA; agarduno3553@sdsu.edu; 2 Division of Epidemiology and Biostatistics, School of Public Health, San Diego State University, San Diego, CA 92182, USA; 3 Moores Cancer Center, School of Medicine, University of California, San Diego, CA 92037, USA

**Keywords:** CYP1A2, liver enzymes, caffeine metabolism, smoking status, smoking intensity, secondhand smoke exposure

## Abstract

Non-alcoholic fatty liver disease (NAFLD) is common among 30% of American adults. Former and current smokers are at higher risk for NAFLD compared to never smokers. The ratio of urine caffeine metabolites to caffeine intake—namely, urine caffeine metabolite indices—has previously been used as a proxy for CYP1A2 activity, which is one of the main liver metabolizing enzymes. CYP1A2 activity is associated with NAFLD progression. No studies to our knowledge have examined the associations of liver enzymes, smoking intensity, and secondhand smoke (SES) with CYP1A2 activity (using caffeine metabolite indices) across smoking status. We analyzed national representative samples from the 2009–2010 National Health and Nutrition Examination Survey (NHANES). Interestingly, even within a normal range, several liver enzymes were associated with caffeine metabolite indices, and patterns of many of these associations varied by smoking status. For instance, within a normal range, aspartate aminotransferase (AST) in never smokers and bilirubin in current smokers were inversely associated with 1-methyluric acid and 5-acetylamino-6-amino-3-methyluracil (URXAMU). Furthermore, we observed a common pattern: across all smoking statuses, higher AST/alanine aminotransferase (AST/ALT) was associated with 1-methyluric acid and URXAMU. Moreover, in current smokers, increased lifelong smoking intensity was associated with reduced caffeine metabolite indices, but acute cigarette exposure as measured by SES levels was associated with increased caffeine metabolite indices among never smokers. In summary, commonly used liver enzyme tests can reflect the CYP1A2 activity even within a normal range, but the selection of these enzymes depends on the smoking status; the associations between smoking and the CYP1A2 activity not only depend on the intensity but also the duration of tobacco exposure.

## 1. Introduction

Non-alcoholic fatty liver disease (NAFLD) is prevalent among 30% of US adults, and is characterized by the presence of fatty liver cells among those without a history of excess alcohol intake, steatogenic medication, or of high genetic risk [1,2,3]. Cigarette smoking is a strong risk factor for worsening liver disease [4,5]. Epidemiological studies have shown that a higher lifelong smoking intensity in current and former smokers is associated with a higher risk of NAFLD [6,7,8]. Yet, diagnosing NAFLD is relatively difficult at the early stages, as there is no consensus on the most appropriate screening measure. Liver biopsy, imaging or sonography are more appropriate for diagnosis as opposed to screening, given their invasiveness and out-of-pocket cost [9]. In addition, the fatty liver index (FLI) is an algorithm that has been used to predict NAFLD, which is based on factors related to obesity and metabolic syndrome [9]. Yet, FLI may not predict the liver function at early-stages of NAFLD, and its predictive ability across individuals with differing smoking histories is unknown.

NAFLD often progresses within a normal range of liver enzymes, especially in early disease stages. High levels of liver enzymes can reflect liver injury. Alanine aminotransferase (ALT), aspartate aminotransferase (AST), alkaline phosphatase (ALP), and/or gamma-glutamyl transpeptidase (GGT) levels in liver parenchyma have been found to be associated with CYP1A2 activities in the rat, the dog, and the human [10]. However, within normal ranges, serum levels of these enzymes have not been used for predicting NAFLD and CYP1A2 activities. Combining these serum liver enzymes with the CYP1A2 activity may better predict the early stage NAFLD than liver enzymes on their own. Few studies have evaluated these relationships in large population studies, especially within a normal range of these liver enzymes.

The ratio of urine caffeine metabolites to caffeine intake—namely, caffeine metabolite indices—has previously been used as a proxy for CYP1A2 activity, which is one of the main liver metabolizing enzymes. Major caffeine metabolites include paraxanthine (82%), followed by theobromine (11%) and theophylline (5%), which are catalyzed by CYP1A2 [6]. Prior studies have measured the CYP1A2 activity using urinary, plasma, and saliva caffeine metabolic indices [8,11,12]. Decreased CYP1A2 activity has been associated with NAFLD progression, and high levels of serum liver enzymes have predicted NAFLD [13,14,15]. A few observational studies have reported that the current smoking intensity was associated with increased CYP1A2 activity in a dose response pattern [12]. In contrast, a smoking cessation intervention study showed that the CYP1A2 activity was reduced after smoking cessation [16,17]. However, this prior study only examined the short-term impact of smoking on CYP1A2, not the long-term impact. No studies to our knowledge have examined the impact of lifelong smoking intensity and secondhand smoke (SES) on CYP1A2 activity. Neither have any studies examined the association of liver enzymes, within a normal range, with the CYP1A2 activity (using caffeine metabolite indices) across never, former, and current smokers.

We propose that there is a biological basis for identifying those at high-risk for NAFLD in asymptotic individuals by using liver enzymes in combination with caffeine metabolic indices. Here, we leverage a large, nationally representative sample from the 2009–2010 National Health and Nutrition Examination Survey (NHANES) to determine associations of liver enzymes with several caffeine metabolite indices in never, former, and current smokers. Moreover, we examine the associations of secondhand smoke (SES) and lifelong smoking intensity with caffeine metabolite indices across the smoking status.

## 2. Materials and Methods

### 2.1. Study Population

Our sample was derived from a nationally representative population that had participated in 2009–2010 NHANES. It is an annual survey on the health and nutritional status among non-institutionalized civilians in the United States. From 2009–2010, a comprehensive profile of urinary caffeine metabolites were analyzed, which had not been done in other years. Of the 10,253 participants interviewed during NHANES 2009–2010, 2714 participants had measurements of caffeine metabolites. A detailed blood profile was performed and included several serum liver enzymes (e.g., GGT), cotinine, and creatinine. We finally selected 1465 participants who were ≥18 years old, had measurements of urinary caffeine metabolites and serum cotinine, and had information on caffeine intakes, smoking intensity, and other covariates, e.g., serum creatinine, which was used for calculating the renal function. The National Centre for Health Statistics Research Ethics Review approved the protocol. The current study was an ancillary study using the de-identified data from the WHEL study. Thus, the exempt IRB was approved by the San Diego State University IRB committee (protocol number: Temp-1286).

### 2.2. Serum Liver Enzymes

Serum ALP was measured by the DxC800 system with a 2-Amino-2-Methyl-1-Propanol (AMP) buffer to look at the rate of change in absorbance. Serum ALT and AST were measured using the DxC800 kinetic rate method, examining the rate of change in absorbance as directly proportional to the ALT activity [18]. Total bilirubin was ascertained using a timed-endpoint Diazo method to measure the change in absorbance, and an enzymatic rate method was used to measure GGT [18].

### 2.3. Smoking Status Assessment

Smoking behaviors were collected using a comprehensive questionnaire. Participants provided a self-report on current and prior cigarette use, age of initiation and cessation of smoking, number of cigarettes smoked/day, and frequency of cigarette use over 30 days. Smoking status was classified into three categories: Never, former, and current smokers based on their answers to the question “ever smoking at least 100 cigarettes in their life”. For instance, they were classified as never smokers if they answered “no”; classified as former smokers if they answered “yes” and “did not smoke at the time of survey”; and were classified as current smokers if they answered “yes” and “still smoke every day or some days at the time of survey”.

### 2.4. Assessment of Lifetime Smoke Intensity

Lifetime smoke intensity (LSI) was the total number of cigarettes smoked over a participant’s lifetime. LSI was set to 0 for never smokers. Separate calculations were done for LSI in the former compared to current smokers, since the former smokers answered a different set of questions as compared to the current smokers. Here, we describe these formulas for LSI: LSI for former smokers = duration of smoking before quitting (years) * (days/year) * (number of cigarettes smoked/day). Smoking duration for former smokers was calculated as the difference between age of quitting and age of initiation. LSI for current smokers = (total days smoked cigarettes/month) * (average cigarettes/day) * (months/year) * duration of smoking (years). Smoking duration for current smokers was calculated as the difference between age at this survey and age of initiating smoking.

### 2.5. Assessment of Serum Cotinine

Secondhand smoke exposure in never and former smokers was assessed via the measurement of serum cotinine (ng/mL), which is a major metabolite of nicotine. In non-smokers, cotinine levels reflect a participant’s environmental exposure to tobacco, whereas in smokers, cotinine levels represent acute exposure from smoking [19,20]. Serum cotinine was measured using isotope-dilution high-performance liquid chromatography/atmospheric pressure chemical ionization tandem mass spectrometric (ID HPLC-APCI MS/MS method). Additional details of the protocol were previously described [21].

### 2.6. Caffeine Intake Assessment

Current caffeine intake was available from a 24-h recall that used a multi-pass method algorithm from the US Department of Agriculture [22]. Trained interviewers asked participants about their food and beverage intake in the past 24 h. The first day of the interview took place in-person at the NHANES mobile examination center (MEC), while the second 24-h recall was conducted over-the-phone 3–10 days after the initial interview [23]. The amount of caffeine consumed was estimated from consumption of all the caffeine-containing foods and beverages. Our analysis primarily focused on Day 1 caffeine intake.

### 2.7. Assessment of Urinary Caffeine Metabolites

**Figure 1:**
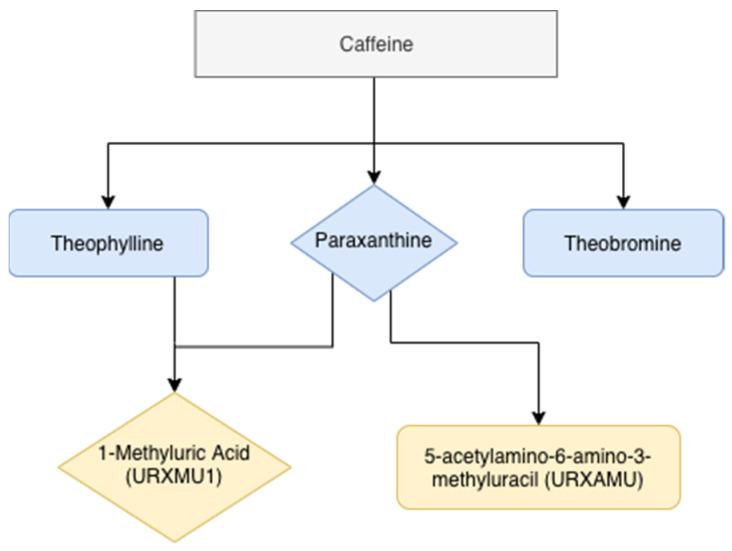
Main caffeine metabolites in humans (adapted from Nehlig et al. [10]).

A total of 14 caffeine metabolites were measured in the NHANES study. We selected the following five metabolites for our analyses: Theophylline, paraxanthine, theobromine, 1-methyluric acid, and 5-acetylamino-6-amino-3-methyluracil (URXAMU), since theophylline, paraxanthine, and theobromine are the main three upstream metabolites, and 1-methyluric acid and URXAMU are the main downstream metabolites that have been shown strongly associated with caffeine clearance [10]. The transformation of these five metabolites are illustrated in Figure 1. Metabolites are quantified using high performance liquid chromatography-electrospray ionization-tandem quadruple mass spectrometry (HPLC-ESI-MS/MS). Detailed methods were described in the NHANES protocol [18]. Urine creatinine was measured using the Roche/Hitachi Modular P Chemistry Analyzer [18]. The urinary concentration of caffeine metabolites (umol/L) is divided by creatine levels, and expressed as (umol/mg). In order to better assess the caffeine metabolism, we created the caffeine metabolite index. The equation is calculated as: Caffeine metabolite index = urine creatinine-adjusted caffeine metabolites/daily self-reported caffeine intake.

### 2.8. Other Assessments

Participants provided information on their demographics, lifestyle habits, and general health. For instance, demographic information included age at screening, race/ethnicity (Non-Hispanic White/Mexican American/Other Hispanic/Non-Hispanic Black/Other Race/Multi-Racial), gender (male/female), and body mass index. The following variables related to blood collection, and health condition were classified as categorical variables, including fasting status (0–8 h, greater than 8 to 12 h, and greater than 12 h), hypertension status (hypotensive, pre-hypertensive, hypertensive, and normotensive), and diabetes (yes/no/borderline).

Physical activity was evaluated using total metabolic equivalents (METs) per week based on a participant’s total amount of moderate and/or vigorous exercise per week. First, total minutes were summed within each exercise intensity category, then multiplied by the respective MET score suggested by NHANES [24]. MET scores increase with respect to the intensity of exercise (e.g., vigorous intensity = 8; moderate intensity = 4; walking/biking for transportation = 4). Physical activity (MET-minutes per week) was derived from the following equation: (Minutes of vigorous activity at work per day) * (days of vigorous work activity) * (MET score = 8) + (minutes of moderate activity at work per day) * (days of moderate work activity)*(MET score = 4) + (minutes of walking/biking transportation per day) * (days of transportation) * (MET score = 4) + (minutes of vigorous recreational activities per day) * (days of vigorous recreational activities)*(MET score = 8) + (minutes of moderate recreational activities per day) * (days of moderate recreational activities) * (MET score = 4). Then, we converted the MET-minutes per week to MET-hours per week. For our data analyses, physical activity was classified into quartiles at 0, 0 > −4, 4 > −40, and <40+ MET-hours per week. 

We calculated the estimated glomerular filtration rate (eGFR) by the Chronic Kidney Disease Epidemiology Collaboration (CKD-Epi) equation model using serum creatinine, age, sex, and race. Details of this equation are previously described [25]. For our data analyses, we categorized eGFR into clinically meaningful groups: Less than 60, 60–90, or greater than 90 mL/min/1.73 m².

### 2.9. Statistical Analyses

All statistical analyses were conducted after accounting for the complex sampling from the survey design. Differences in participant characteristics (e.g., age, race, and body mass index) between smoking groups were evaluated using chi-square tests. We conducted these tests using the Rao-Scott chi-square rather than the Wald chi-square, in order to avoid overestimating between-group differences. To examine how smoking groups differed in terms of their liver enzyme levels, we then classified liver enzymes into quartiles and reported participant counts broken up by these quartiles. To further consider clinically meaningful cut-points, we further split quartile 1 and 4 into normal and abnormal enzyme levels, based on the American Board of Internal Medicine Laboratory Test 2020 guidelines’ definition of abnormal liver enzyme levels [26]. In other words, quartiles were restricted to normal enzyme levels. For instance, the ALP abnormal range was defined as 30 U/L; ≥40 U/L for ALT and AST; ≥1 U/L for bilirubin. A gender-specific cut-point was used for GGT: ≥40 U/L for women and ≥40 U/L for men.

Linear regression was used to model associations between liver enzyme levels and caffeine metabolite indices, where these caffeine indices were proxies for CYP1A2 activity. Each caffeine metabolite index was log-transformed to maintain its normal distribution. Liver enzymes were characterized by quartiles plus additional abnormal categories as stated above. Covariate selection was based on *a priori* hypotheses from reviewing the literature. Models were adjusted for hypertension status, gender, ethnicity, body mass index, diabetes, liver condition, fasting status, physical activity, and eGFR. In addition, liver enzymes were adjusted simultaneously in the multivariate models with the exception that AST and ALT were not adjusted simultaneously, since AST was highly correlated with ALT (r = 0.71). For instance, models containing AST are simultaneously adjusted for all the liver enzymes except for ALT. Stratified analyses by smoking status were conducted to characterize the association of liver enzymes with each caffeine metabolite index within each smoking strata. Secondhand smoking (measured by serum cotinine) and lifestyle smoking intensity were also evaluated as the primary exposure variable. Moreover, we examined their associations with caffeine metabolite indices in the multivariable model.

All the analyses were conducted in SAS 9.4 (SAS Institute, Cary, NC, USA). To account for the complex sampling mentioned above, we used the “PROC SURVEYREG” procedures with the “strata” statement representing strata (sdmvstra), the “cluster” statement representing the sampling unit (sdmvpsu), and the weight statement representing the 2-year sub-sample weight (WTSC2YR). The 2-year subsample weight (WTSC2YR) reflects the smallest denominator of all the covariates sampled. This survey procedure allowed us to represent the sampled population in the US and the response rate. The domain statement was used to distinguish participants who met the inclusion criteria and/or to perform stratified analyses based on smoking status.

## 3. Results

### 3.1. Demographic Characteristics by Smoking Status 

Table 1A displays participant lifestyle and demographic characteristics stratified by smoking status. Never smokers were more often female, Mexican American, non-diabetic, and had higher levels of physical activity. Former smokers were more often males, older than 48, non-Hispanic whites, overweight or obese, diabetic, and pre-hypertensive. Non-Hispanic Blacks, young to middle-aged (18–38) or elderly (68+) adults, and those with the highest lifetime smoking intensity were more likely to be current smokers. Serum cotinine was noticeably higher in current smokers, followed by former and never smokers. Current smokers were more likely to have reduced renal function with eGFR < 60 (74.9% in current smokers vs. 65% in never and 59% in former smokers).

### 3.2. Liver Enzyme Levels by Smoking Status in All the Participants in This Study

Table 1B displays liver enzyme levels across all smoking groups. Never smokers had the lowest liver enzyme levels as compared to other smoking groups, except for bilirubin (higher than the other two groups), whereas former smokers were more likely to have elevated AST, ALT, and bilirubin. Current smokers were more likely to have bilirubin levels in the first quartile (26% in current vs. 17% in former and 18% in never smokers). The AST/ALT ratio did not significantly differ across the three smoking strata.

### 3.3. The Associations between Liver Enzymes and Day 1 Caffeine Metabolite Indices

Table 2 presents the associations between serum liver enzymes and each caffeine metabolite index, where the index was based on caffeine intake at day 1. The estimates shown in this table are multivariable-adjusted. Overall, most liver enzymes had differential patterns with caffeine metabolite indices across smoking strata. Results for each liver enzyme are described separately in the paragraphs below.

ALP: Never and current smokers had reversed trends between the ALP enzyme and two upstream metabolite indices (theophylline and paraxanthine). For instance, within normal liver enzyme levels, never smokers had a negative association between ALP and paraxanthine, where an increase in ALP grouping (quartile 4 relative to quartile 1) was associated with a decrease in the paraxanthine index (beta = −0.23; *p*-value for trend = 0.08), whereas this comparison among current smokers was associated with an increase in the paraxanthine index (beta = 0.59; *p*-value for trend = 0.007). ALP was positively associated with all of the caffeine metabolite indices in current smokers (*p*-value for trend: 0.007 to 0.07). In former smokers, a positive association was observed between ALP and two downstream metabolite indices (1-methyluric acid and URXAMU). These relationships in former smokers appeared to be bell-shaped and were significant at the 0.05 level.

AST: AST was inversely associated with all caffeine metabolite indices among never smokers, where the *p*-value for trends were either statistically significant (*p* < 0.05) or marginally significant (*p* ≤ 0.12). There was evidence of a non-linear relationship between AST and caffeine metabolite indices in former and current smokers: increasing levels of AST were associated with an initial increase followed by a decline in caffeine metabolite indices among former and current smokers.

ALT: ALT enzyme was not associated with any caffeine metabolite indices among any smoking groups. Note, there was a marginal trend among former smokers. Specifically, an inverse association was shown between ALT and all three upstream metabolite indices (theophylline, paraxanthine, and theobromine).

AST/ALT Ratio: There were inverse associations between the AST/ALT ratio and the two downstream metabolite indices (1-methyluric acid and URXAMU) in all the smoking groups, though some were statistically significant and some were marginally significant (*p* for trend ≤ 0.1).

Bilirubin: Among never and former smokers, there were no clear patterns of association between bilirubin and caffeine metabolite indices. Yet, we observed strong and reverse associations in current smokers for the two downstream metabolite indices. For instance, for the 1-methyluric acid index, compared to the reference group (quartile 1), the beta estimates for people with bilirubin at quartiles 2, 3, 4, and above a normal range were −0.51, −1.09, −0.99, and −1.07, respectively (*p*-value for trend = 0.002).

GGT: We found positive associations between GGT and several caffeine metabolite indices among current smokers, although no obvious patterns were seen among never or former smokers. For instance, in former smokers, we observed positive and linear associations between GGT and all the caffeine metabolite indices (*p*-value for trend ≤0.07 for all). In never smokers, we observed a positive and linear association between GGT and most of the metabolite indices (*p*-value for trend ≤0.07), except for theobromine.

**Figure 2:**
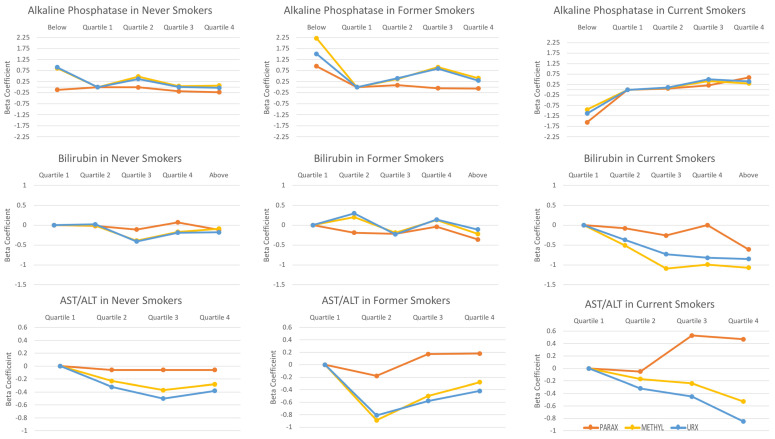
Patterns of selected liver enzyme levels with some caffeine metabolite indices in never, former, and current smokers. The covariates adjusted in multivariable models included age, gender, ethnicity, body mass index, physical activity, diabetes, hypertension status, liver condition, glomerular filtration rate, fasting status, serum cotinine, and total smoking intensity. PARAX: Paraxanthine; METHYL: 1-methyluric acid; URX: 5-acetylamino-6-amino-3-methyluracil.

Summary figure: A summary figure illustrating the associations of ALP, Bilirubin, and AST/ALT ratio with the selected caffeine metabolite indices were presented in Figure 2.

### 3.4. The Associations between Liver Enzymes and Day 2 Caffeine Metabolites

The associations between liver enzymes and day 2 caffeine metabolites were similar. Yet, the results were less consistent when relying on statistical testing, compared to the results using caffeine metabolite indices based on the self-report from day 1 (Table S1). 

### 3.5. The Associations between Serum Cotinine and Smoking Intensity and Caffeine Metabolite Indices

Serum cotinine levels: Serum cotinine was positively associated with two downstream metabolite indices (1-methyluric acid and URXAMU) in never (*p*-value for trend ≤0.05) and current smokers (≤0.03) but inversely associated with the same two indices in former smokers (*p*-value > 0.05, Table 3). Among never smokers, cotinine was inversely associated with the theobromine index (*p*-value for trend = 0.03).

Lifelong Smoking intensity: We assessed lifelong former smoking intensity for former smokers and lifelong smoking intensity (from initiation to the present) for current smokers (Table 3). The associations between smoking intensity and caffeine metabolite indices had opposite patterns when comparing former to current smokers. Increased smoking intensity was associated with increased levels of the two upstream metabolite indices (theophylline and paraxanthine) in former smokers (*p*-value for trend ≤0.09) but decreased levels of the same two metabolite indices in current smokers (*p*-value for trend ≤0.006). In current smokers, in addition to the upstream metabolite indices, high smoking intensity was also inversely associated with all downstream metabolite (1-methyluric acid and URXAMU) indices (*p* ≤ 0.13). Notably, in former smokers, even though we observed a positive association between former smoking intensity and caffeine metabolite indices, the association was bell-shaped rather than linear. 

## 4. Discussion

In a nationally representative sample, our study is the first to identify key liver enzymes associated with caffeine metabolite indices (a proxy for CYP1A2 activity), and whether these associations vary by smoke status, in order to determine lifelong smoking intensity and short-term tobacco exposure (i.e., SES) with caffeine metabolite indices. Even within normal liver enzyme levels, several liver enzymes were associated with reduced caffeine metabolite indices. Increased AST/ALT ratio was associated with reduced caffeine metabolite indices across all smoking statuses. Higher ALP and AST levels in never smokers and higher bilirubin levels in current smokers were associated with lower caffeine metabolite indices. In current smokers, increased lifelong smoking intensity was associated with reduced caffeine metabolite indices, but acute cigarette exposure, as measured by serum cotinine, was associated with increased caffeine metabolite indices. This finding may partially explain a key observation: long-term smoking can increase the risk of chronic liver diseases, but short-term cigarette smoking can also temporarily increase liver activity [12,17].

Prior studies have shown that liver enzymes levels are not indicative of NAFLD on their own as changes of these enzymes may also reflect other health conditions. For example, AST and ALT were associated with hepatocyte injury or fat accumulation in the liver [27]. Yet, higher AST and ALT levels were also associated with rhabdomyolysis and hemolysis [27]. Bilirubin is a liver enzyme involving heme catabolism but can also regulate cholesterol metabolism, adipokines, and contributes to insulin sensitivity [28]. Higher GGT levels may be due to liver damage or diabetes [29]. Conversely, normal liver enzyme levels (e.g., AST and ALT) do not guarantee the absence of NAFLD, non-alcoholic steatohepatitis (NASH), advanced cirrhosis, and fibrosis [27,30]. Thus, it is important to evaluate how these liver enzymes are associated with caffeine metabolite indices within normal levels of these enzymes, since these indices have long been used to study the liver function and activity (i.e., CYP1A2 activity).

Using both liver enzyme and caffeine metabolite indices, our study indicates potential implications of these liver enzymes—even within a normal range, changes of these enzymes may reflect altered CYP1A2 activity. We identified several specific patterns and a common pattern between liver enzymes and caffeine metabolite indices. For instance, we found that high levels of ALP and AST in never smokers and higher levels of bilirubin in current smokers were associated with reduced CYP1A2 activity. We also detected a common pattern across three groups: AST/ALT ratio was inversely associated with two downstream metabolite indices (1-methyluric acid and URXAMU) in never, former, and current smokers. Our study is consistent with a prior study which demonstrated that a higher AST/ALT ratio was associated with more advanced stages of NAFLD, even among those with normal aminotransferase levels [31]. Even within normal ranges, these liver enzymes may reflect early liver damage in never, past, and current smokers. Thus, we may use these liver enzymes as an early-stage liver biomarker when caffeine metabolite indices cannot be measured. Most of our findings were consistent with our hypothesis that higher levels of liver enzymes may indicate liver damage except in the case of GGT. Higher GGT was associated with higher caffeine metabolite indices in never and former smokers. A potential explanation for this positive relationship is that the liver may not only be damaged but also be under an excess workload. The liver may try to compensate for this excess workload by increasing levels of liver enzymes to improve its activity.

Smoking intensity and secondhand smoke are both known to be strong risk factors for NAFLD [6,7,8]. Smoking has been found to increase CYP1A2 activity in the short-term [10,12], whereas lifelong smoking intensity was inversely associated with CYP1A2 activity in this current study. The following may help explain this observation. We conjecture that smoking may increase CYP1A2 activity in the short-term but reduce CYP1A2 activity in the long-term. Our findings with serum cotinine, a measure of current smoke intensity, indirectly support our conjectures that short-term or acute cigarette exposure does increase CYP1A2 activity. Furthermore, lifelong smoking intensity, which represents long-term smoking, was associated with reduced CYP1A2 activity. Tobacco smoke contains dioxins and polycyclic aromatic hydrocarbons (PAHs) that activate the aryl hydrocarbon receptor (AHR) and mediate at least part of their toxicity through AHR signaling [32]. AHR promotes induction of CYP1A2, amplifies the adverse effects, and promotes pathogenesis [32,33]. While the degree of affinity to AHR and activation of CYP1A2 activity may vary by individual [32,33], in general, short-term tobacco exposure has been found to activate AHR and increase the CYP1A2 activity [32,33]. Yet, molecular mechanistic studies on the impact of lifelong smoking intensity are limited. Whether the inverse association is associated with reduced affinity of AHR or saturation of CYP1A2 activity after long-term tobacco exposure will need to be studied in the future. Nevertheless, this pathological process may be similar to the regulation of insulin in humans: initially, our pancreatic beta cells produce more insulin during the early stages of insulin resistance, but beta cells are gradually exhausted and can no longer produce insulin at the end stage of diabetes. The initial stage is a self-defense response, but this defense system will be eventually exhausted with the worsening of the disease.

Our study is limited in that the design is cross-sectional, which prevents the interpretation of the associations between liver enzymes and caffeine metabolism as casual. Longitudinal studies are needed to confirm whether these patterns of associations remain over long-term follow up. Reverse causation is another concern with cross-sectional studies, thus the positive or negative associations in these strata will need to be further confirmed in longitudinal studies. Another limitation of this work is that we do not fully control for all possible acute and chronic diseases that may elevate ALP levels, so it is possible that these associations may also be confounded by the presence of other diseases, such as congestive heart failure, benign bone disease, and hyperthyroidism [34]. In our multivariable-adjusted analyses, we did control for the presence of any known liver disease, kidney function, and hypertension status. Yet, there is potential for residual confounding due to the undiagnosed liver disease, such as hepatocellular carcinoma [35,36,37]. Longitudinal studies with access to detailed medical history are needed to confirm our findings.

There are numerous strengths of this study, including that NHANES allowed for the estimation of lifelong smoking intensity, as well as information on caffeine metabolites on a nationally representative population. Prior pharmacologic studies on caffeine metabolism were often comprised of around 20 participants, in contrast to our study which includes over 1000 participants [38]. Moreover, our study focused on a healthy population before the onset of NAFLD and will provide valuable information for studying early-stage liver damage. Moreover, we assessed these associations within a normal range rather than abnormal ranges of these liver enzymes.

## 5. Conclusions

Using non-invasive liver enzyme tests, we are the first study to identify individuals with reduced liver CYP1A2 activity as evidenced by reduced caffeine metabolite indices, among people with normal levels of liver enzymes. High levels of ALP and AST may identify those with reduced liver function in never smokers, whereas bilirubin may identify high-risk groups among current smokers. An increased AST/ALT ratio may predict lower liver function among all participants regardless of their smoking status. Our study may help explain a novel hypothesis that long-term and high intensity smoking may decrease but short-term smoking may increase the CYP1A2 activity. Longitudinal studies with detailed measures on liver conditions and comorbidities that may modify liver enzymes and CYP1A2 activity are needed.

## Figures and Tables

**Table 1:** (**A**) Demographic characteristics in selected 2009–2010 NHANES participants (*n* = 1465); (**B**) liver enzyme levels by smoking status in the selected NHANES 2009–2010 participants (*n* = 1465).

(A)				
Characteristic	Never Smoker (<100 cig. Ever) (N = 812)	Former Smoker (>100 cig. Ever) (N = 348)	Current Smoker (>100 cig. Ever) (N = 305)	*p*-Value
Gender (N, %)				≤0.0001
Female	489 (56.95)	136 (39.98)	146 (47.19)	
Male	323 (43.05)	212 (60.02)	159 (52.81)	
Age (N, %)				≤0.0001
18 to <28	136 (17.59)	30 (11.53)	61 (23.81)	
28 to <38	158 (22.35)	46 (16.61)	66 (21.93)	
38 to <48	127 (17.92)	70 (23.32)	61 (18.43)	
48 to <58	131 (14.56)	85 (24.72)	45 (12.04)	
58 to <68	147 (12.51)	105 (19.08)	26 (5.54)	
68+	113 (15.08)	12 (4.74)	46 (18.26)	
Ethnicity (N, %)				0.01
Mexican American	175 (9.82)	49 (5.24)	47 (7.86)	
Other Hispanic	95 (5.44)	36 (4.29)	22 (3.37)	
Non-Hispanic Black	120 (8.97)	32 (4.89)	61 (12.90)	
Other Race/Multiracial	42 (6.12)	15 (5.83)	11 (5.50)	
Non-Hispanic White	380 (69.65)	216 (79.75)	164 (70.37)	
Serum cotinine, ng/mL, (N, %)				<0.0001
≤1.7 × 10^−6^	269 (34.86)	90 (25.50)	1 (0.21)	
1.7 × 10^−6^ > to 5.20 × 10^−6^	290 (36.08)	110 (32.83)	1 (0.15)	
5.2 × 10^-6^ > to 113.00 × 10^−6^	198 (22.27)	98 (26.81)	15 (3.43)	
>113.0 × 10^−6^	55 (6.80)	50 (14.86)	288 (96.21)	
Smoking intensity, total lifetime cigarettes, (N, %)				NA
0	812 (100.00)	NA	NA	
0 > to 50,400	NA	152 (45.29)	122 (38.90)	
50,400+	NA	196 (54.72)	183 (61.11)	
Body Mass Index, kg/m^2^, (N, %)				0.04
<18.5	8 (1.13)	3 (1.20)	7 (3.98)	
18.5–25	222 (30.00)	73 (25.04)	92 (29.99)	
25–30	251 (30.42)	139 (35.70)	100 (34.94)	
30+	331 (38.44)	133 (38.06)	106 (31.09)	
Blood pressure status (N, %)				0.02
Hypotensive	6 (0.85)	2 (0.30)	3 (1.02)	
Normotensive	384 (52.39)	124 (38.29)	151 (52.47)	
Pre-Hypertensive	278 (33.46)	148 (45.19)	104 (34.83)	
Hypertensive	144 (13.29)	74 (16.23)	47 (11.68)	
Diabetes (N, %)				<0.0001
Yes	79 (5.99)	55 (13.67)	20 (5.88)	
Borderline	11 (1.00)	11 (4.08)	11 (2.63)	
No	722 (93.01)	282 (82.25)	274 (91.49)	
Fasting Status, hours (N, %)				0.56
0–8	415 (53.63)	180 (49.91)	159 (53.47)	
8–12	187 (23.33)	76 (24.13)	86 (26.82)	
12+	210 (23.05)	92 (25.96)	60 (19.71)	
Liver Condition (N, %)				0.20
Former	14 (1.26)	6 (1.37)	5 (3.12)	
Current	6 (0.43)	5 (1.37)	4 (0.84)	
No Liver Condition	792 (98.31)	337 (97.26)	296 (96.04)	
Estimated Glomerular Filtration Rate, mL/min/1.73 cm^2^ (N, %)				0.0002
Less than 60	528 (65.51)	179 (59.20)	223 (74.93)	
60–90	237 (30.65)	126 (32.46)	75 (23.16)	
Greater than 90	47 (3.84)	43 (8.34)	7 (1.91)	
Physical activity, metabolic equivalent-hours/week, (N, %)				0.02
0	220 (21.57)	89 (22.13)	89 (24.39)	
0>−4	48 (4.97)	30 (8.66)	15 (3.68)	
4>−40	323 (42.91)	115 (32.89)	92 (33.18)	
40+	221 (30.56)	114 (36.32)	109 (38.75)	
**(B)**				
Alkaline Phosphatase, U/L, (N, %)				0.03
<30.00 (Abnormal)	6 (0.96)	1 (0.07)	2 (0.28)	
30.00 to <55.29	234 (33.06)	85 (29.26)	61 (22.03)	
55.29 to < 68.64	209 (25.29)	101 (26.25)	87 (29.29)	
68.64 to <86.05	207 (24.25)	93 (27.47)	94 (32.01)	
≥86.05	156 (16.44)	68 (16.96)	61 (16.39)	
Alanine Aminotransferase, U/L, (N, %)				0.06
<15.34	155 (17.87)	47 (10.88)	69 (21.36)	
15.34 to <19.70	204 (24.84)	70 (20.58)	71 (21.43)	
19.73 to <27.09	247 (30.29)	125 (36.50)	87 (28.48)	
27.09 to <40.00	126 (17.02)	74 (21.40)	49 (17.15)	
≥40.00 (Abnormal)	80 (9.98)	32 (10.64)	29 (11.58)	
Aspartate Aminotransferase, U/L, (N, %)				
<19.46	176 (19.90)	60 (16.48)	94 (29.74)	0.0003
19.46 to <22.85	165 (21.47)	63 (17.18)	64 (22.11)	
22.8 to <27.43	240 (30.24)	116 (34.02)	66 (21.02)	
27.43 to <40.00	190 (24.14)	94 (29.34)	62 (20.44)	
≥40.00 (Abnormal)	41 (4.25)	15 (2.97)	19 (6.68)	
Aspartate Aminotransferase/Alanine Aminotransferase (N, %)				0.09
<0.92	212 (26.11)	101 (29.02)	90 (29.51)	
0.92 to <1.14	211 (25.99)	100 (28.74)	77 (25.25)	
1.14 to <1.37	232 (28.57)	83 (23.85)	72 (23.61)	
≥1.37	157 (19.33)	64 (18.39)	66 (21.64)	
Bilirubin, U/L, (N, %)				<0.0001
<0.52	165 (18.30)	66 (16.73)	88 (26.19)	
0.52 to <0.60	139 (17.36)	61 (17.20)	80 (27.91)	
0.650 to <0.82	273 (32.85)	137 (39.17)	91 (30.59)	
0.82 to <1.00	134 (17.55)	45 (14.46)	26 (6.85)	
≥1.00 (abnormal)	101 (13.94)	39 (12.44)	20 (8.47)	
Gamma-glutamyl transpeptidase, U/L				
Female (N, %))				0.17
<12.55	133 (33.88)	26 (23.32)	31 (22.44)	
12.55 to <17.94	140 (27.61)	39 (33.05)	36 (26.35)	
17.94 to <27.71	118 (23.49)	34 (22.27)	42 (28.71)	
27.71–40.00	67 (9.98)	17 (11.13)	20 (13.94)	
≥40.00 (Abnormal)	31 (5.04)	20 (10.23)	17 (8.56)	
Male (N, %))				0.73
<12.55	33 (10.04)	18 (7.81)	13 (7.51)	
12.55 to <17.94	65 (20.00)	51 (25.13)	23 (16.33)	
17.94 to <27.71	103 (33.36)	71 (32.62)	53 (37.14)	
27.71–50.00	82 (25.82)	55 (25.82)	44 (26.85)	
≥50.00 (Abnormal)	40 (10.78)	17 (8.62)	26 (12.18)	

Data in each column are presented as the total number (%). NHANES: National Health and Nutrition Examination Survey; cig: Cigarettes.

**Table 2:** Serum liver enzymes and caffeine metabolite indices (caffeine intake was calculated from the 24-h recall on day 1).

		Never Smoker					Former Smoker					Current Smoker				
		Log (Theophylline)/Caffeine	Log (Paraxanthine)/Caffeine	Log (Theobromine)/Caffeine	Log (1-methyluric acid^**^)/Caffeine	Log(URXAMU)/Caffeine	Log (Theophylline)/Caffeine	Log (Paraxanthine)/Caffeine	Log (Theobromine)/Caffeine	Log (1-methyluric acid)/Caffeine	Log (URXAMU)/Caffeine	Log (Theophylline)/Caffeine	Log (Paraxanthine)/Caffeine	Log (Theobromine)/Caffeine	Log (1-methyluric acid^**^)/Caffeine	Log (URXAMU)/Caffeine
		Beta (P)	Beta (P)	Beta (P)	Beta (P)	Beta (P)	Beta (P)	Beta (P)	Beta (P)	Beta (P)	Beta (P)	Beta (P)	Beta (P)	Beta (P)	Beta (P)	Beta (P)
**Alkaline Phosphatase (U/L)**																
Abnormal	<30	−0.16 (0.70)	−0.12 (0.77)	−0.67 (0.25)	0.86 (0.22)	0.9 (0.14)	1.24 (0.04)	0.95 (0.12)	0.03 (0.98)	2.22 (0.04)	1.51 (0.18)	−1.77 (0.07)	−1.56 (0.11)	−2.04 (0.08)	−0.95 (0.40)	−1.13 (0.26)
Q1	30 to <55.29	Ref	Ref	Ref	Ref	Ref	Ref	Ref	Ref	Ref	Ref	Ref	Ref	Ref	Ref	Ref
Q2	55.29 to < 68.64	0.02 (0.83)	−0.01 (0.91)	0.33 (0.04)	0.48 (0.04)	0.37 (0.15)	−0.02 (0.91)	0.09 (0.70)	0.17 (0.52)	0.37 (0.15)	0.4 (0.17)	0.13 (0.44)	0.06 (0.74)	−0.07 (0.79)	0.10 (0.80)	0.11 (0.80)
Q3	68.64 to <86.05	−0.18 (0.31)	−0.19 (0.32)	0.11 (0.60)	0.05 (0.84)	0.004 (0.99)	−0.09 (0.66)	−0.05 (0.83)	−0.01 (0.98)	0.91 (<0.0001)	0.84 (<0.0001)	0.23 (0.14)	0.21 (0.27)	−0.04 (0.84)	0.41 (0.11)	0.50 (0.09)
Q4	≥86.05	−0.21 (0.15)	−0.23 (0.10)	0.10 (0.52)	0.07 (0.79)	−0.03 (0.91)	−0.1 (0.67)	−0.06 (0.82)	−0.20 (0.51)	0.41 (0.17)	0.30 (0.25)	0.40 (0.06)	0.59 (0.01)	0.47 (0.055)	0.30 (0.39)	0.40 (0.35)
**P for trend**		0.12	0.08	0.58	0.84	0.64	0.47	0.58	0.44	0.01	0.03	0.03	0.007	0.06	0.07	0.07
**Aspartate Aminotransferase (U/L)**																
Q1	<19.46	Ref	Ref	Ref	Ref	Ref	Ref	Ref	Ref	Ref	Ref	Ref	Ref	Ref	Ref	Ref
Q2	19.46 to <22.85	−0.32 (0.06)	−0.31 (0.11)	−0.58 (0.01)	−0.12 (0.68)	−0.31 (0.26)	0.26 (0.24)	0.13 (0.56)	−0.04 (0.89)	0.28 (0.39)	0.21 (0.42)	−0.17 (0.42)	−0.21 (0.43)	−0.33 (0.16)	−0.96 (0.01)	−1.00 (0.01)
Q3	22.8 to <27.43	−0.24 (0.11)	−0.26 (0.11)	−0.67 (0.004)	−0.28 (0.12)	−0.33 (0.14)	0.39 (0.04)	0.38 (0.03)	0.03 (0.90)	−0.20 (0.38)	−0.19 (0.51)	0.45 (0.12)	0.33 (0.32)	0.43 (0.26)	−0.15 (0.63)	−0.34 (0.29)
Q4	27.43 to <40	−0.33 (0.07)	−0.33 (0.13)	−0.57 (0.02)	−0.50 (0.004)	−0.65 (0.001)	−0.03 (0.78)	−0.01 (0.95)	−0.07 (0.80)	−0.22 (0.42)	−0.06 (0.82)	0.28 (0.33)	0.32 (0.30)	0.27 (0.50)	−0.44 (0.41)	−0.52 (0.38)
Abnormal	≥40	−0.25 (0.32)	−0.47 (0.09)	−0.53 (0.07)	−0.46 (0.19)	−0.54 (0.14)	−0.67 (0.10)	−0.8 (0.06)	−1.16 (0.01)	−0.14 (0.71)	−0.29 (0.45)	−0.6 (0.07)	−0.49 (0.18)	−0.59 (0.10)	−0.23 (0.62)	−0.42 (0.33)
**P for trend**		0.12	0.09	0.02	0.008	0.005	0.32	0.31	0.42	0.29	0.55	0.44	0.41	0.57	0.60	0.41
**Alanine Aminotransferase (U/L)**																
Q1	<15.34	Ref	Ref	Ref	Ref	Ref	Ref	Ref	Ref	Ref	Ref	Ref	Ref	Ref	Ref	Ref
Q2	15.34 to <19.73	−0.14 (0.32)	−0.09 (0.52)	−0.22 (0.30)	−0.17 (0.28)	−0.15 (0.42)	−0.65 (0.04)	−0.47 (0.07)	−0.58 (0.11)	−0.46 (0.31)	−0.26 (0.52)	0.32 (0.06)	0.30 (0.12)	0.35 (0.20)	−0.18 (0.62)	−0.33 (0.39)
Q3	19.73 to <27.09	−0.09 (0.49)	−0.08 (0.56)	−0.32 (0.13)	−0.26 (0.19)	−0.19 (0.41)	−0.39 (0.02)	−0.26 (0.20)	−0.54 (0.19)	−1.04 (0.004)	−0.85 (0.02)	0.24 (0.33)	0.19 (0.44)	0.28 (0.47)	0.46 (0.19)	0.44 (0.20)
Q4	27.09 to <40	−0.35 (0.052)	−0.30 (0.13)	−0.53 (0.03)	−0.33 (0.14)	−0.27 (0.25)	−0.60 (0.04)	−0.41 (0.19)	−0.63 (0.19)	−0.39 (0.33)	0.01 (0.97)	0.16 (0.68)	0.06 (0.89)	0.58 (0.28)	0.36 (0.37)	0.34 (0.42)
Abnormal	≥40	−0.17 (0.55)	−0.08 (0.78)	−0.07 (0.83)	0.10 (0.56)	−0.15 (0.42)	−0.73 (0.07)	−0.59 (0.18)	−0.60 (0.15)	−0.32 (0.37)	−0.03 (0.93)	−0.24 (0.57)	−0.07 (0.87)	0.07 (0.87)	0.005 (0.99)	0.05 (0.90)
**P for trend**		0.33	0.48	0.30	0.55	0.54	0.25	0.37	0.28	0.67	0.50	0.69	0.75	0.56	0.42	0.32
**Aspartate/Alanine Aminotransferase**																
Q1	<0.92	Ref	Ref	Ref	Ref	Ref	Ref	Ref	Ref	Ref	Ref	Ref	Ref	Ref	Ref	Ref
Q2	0.92 to <1.14	0.02 (0.88)	−0.06 (0.64)	−0.16 (0.40)	−0.23 (0.32)	−0.32 (0.17)	−0.22 (0.15)	−0.18 (0.24)	−0.24 (0.10)	−0.89 (0.02)	−0.81 (0.03)	−0.09 (0.64)	−0.05 (0.82)	−0.51 (0.03)	−0.17 (0.47)	−0.32 (0.26)
Q3	1.14 to <1.37	0.06 (0.69)	−0.06 (0.68)	0.12 (0.63)	−0.37 (0.09)	−0.50 (0.03)	0.12 (0.70)	0.17 (0.61)	0.24 (0.36)	−0.50 (0.03)	−0.58 (0.005)	0.49 (0.06)	0.53 (0.06)	0.24 (0.30)	−0.24 (0.36)	−0.45 (0.13)
Q4	≥1.37	0.08 (0.59)	−0.06 (0.70)	−0.11 (0.61)	−0.28 (0.19)	−0.38 (0.11)	0.22 (0.39)	0.18 (0.50)	0.10 (0.73)	−0.28 (0.25)	−0.42 (0.10)	0.45 (0.08)	0.47 (0.14)	−0.09 (0.74)	−0.53 (0.18)	−0.85 (0.04)
**P for trend**		0.56	0.71	0.33	0.20	0.07	0.36	0.41	0.21	0.045	0.0015	0.02	0.06	0.99	0.16	0.02
**Bilirubin (U/L)**																
Q1	<0.52	Ref	Ref	Ref	Ref	Ref	Ref	Ref	Ref	Ref	Ref	Ref	Ref	Ref	Ref	Ref
Q2	0.52 to <0.650	−0.03 (0.78)	−0.02 (0.87)	−0.24 (0.15)	−0.01 (0.98)	0.02 (0.95)	−0.05 (0.84)	−0.19 (0.45)	-0.18 (0.51)	0.20 (0.53)	0.30 (0.45)	-0.13 (0.61)	-0.08 (0.74)	0.10 (0.77)	-0.51 (0.049)	-0.37 (0.13)
Q3	0.650 to <0.82	−0.11 (0.45)	−0.11 (0.54)	−0.47 (0.02)	−0.39 (0.10)	−0.41 (0.10)	−0.22 (0.38)	−0.22 (0.43)	−0.20 (0.50)	−0.19 (0.54)	−0.23 (0.56)	−0.14 (0.42)	−0.26 (0.11)	−0.31 (0.27)	−1.09 (0.0003)	−0.73 (0.01)
Q4	0.82 to <1	−0.02 (0.90)	0.07 (0.69)	−0.30 (0.14)	−0.17 (0.38)	−0.19 (0.43)	0.05 (0.86)	−0.04 (0.91)	−0.28 (0.39)	0.13 (0.78)	0.14 (0.75)	0.14 (0.61)	0.001 (0.998)	−0.10 (0.81)	−0.99 (0.02)	−0.82 (0.03)
Abnormal	≥1	−0.14 (0.42)	−0.12 (0.53)	−0.18 (0.52)	−0.09 (0.64)	−0.18 (0.47)	−0.40 (0.16)	−0.36 (0.27)	−0.60 (0.15)	−0.22 (0.66)	−0.11 (0.86)	−0.25 (0.48)	−0.61 (0.06)	−0.74 (0.03)	−1.07 (0.02)	−0.85 (0.04)
**P for trend**		0.56	0.82	0.37	0.41	0.29	0.32	0.50	0.24	0.63	0.72	0.68	0.09	0.22	0.002	0.02
**Gamma-glutamyl transpeptidase (U/L)**																
Q1	<12.55	Ref	Ref	Ref	Ref	Ref	Ref	Ref	Ref	Ref	Ref	Ref	Ref	Ref	Ref	Ref
Q2	12.55 to <17.94	0.22 (0.25)	0.15 (0.41)	0.14 (0.60)	0.04 (0.90)	0.06 (0.83)	0.23 (0.23)	0.23 (0.31)	0.79 (0.01)	0.17 (0.59)	0.51 (0.15)	−0.32 (0.18)	−0.34 (0.14)	0.25 (0.44)	−0.26 (0.51)	−0.23 (0.56)
Q3	17.94 to <27.71	0.08 (0.53)	−0.00004 (0.9997)	−0.17 (0.44)	−0.02 (0.94)	−0.01 (0.94)	0.31 (0.06)	0.23 (0.29)	0.95 (0.01)	0.30 (0.32)	0.60 (0.11)	−0.30 (0.02)	−0.38 (0.02)	0.03 (0.89)	−0.23 (0.59)	−0.22 (0.59)
Q4	Female: 27.71–40; Male: 27.71−50	0.48 (0.003)	0.36 (0.02)	0.26 (0.27)	0.47 (0.07)	0.56 (0.04)	0.48 (0.04)	0.43 (0.08)	0.96 (0.01)	0.83 (0.01)	0.93 (0.02)	0.07 (0.78)	−0.08 (0.74)	0.35 (0.23)	0.55 (0.33)	0.44 (0.41)
Abnormal	Female: ≥40; Male: ≥50	0.45 (0.03)	0.30 (0.12)	−0.08 (0.78)	0.26 (0.43)	0.35 (0.23)	0.65 (0.01)	0.43 (0.07)	0.81 (0.01)	0.27 (0.38)	0.91 (0.03)	0.43 (0.17)	0.09 (0.79)	−0.13 (0.78)	0.46 (0.44)	0.37 (0.47)
**P for trend**		0.01	0.08	0.94	0.10	0.04	0.02	0.07	0.01	0.07	0.04	0.24	0.99	0.95	0.21	0.245

We used the day 1 caffeine intake to calculate caffeine metabolite indices. The covariates adjusted in multivariable models included age, gender, ethnicity, body mass index, physical activity, diabetes, hypertension status, liver condition, glomerular filtration rate, fasting status, serum cotinine, and total smoking intensity. Total smoking intensity is calculated separately for former and current smokers. URXAMU: 5-acetylamino-6-amino-3-methyluracil.

**Table 3:** Secondhand smoke and smoking intensity in relation to caffeine metabolite indices (caffeine intakes was calculated from the 24-h recall on day 1).

		Never Smoker					Former Smoker					Current Smoker				
		Log (Theophylline)/Caffeine	Log (Paraxanthine)/Caffeine	Log (Theobromine)/Caffeine	Log (1-methyluric acid)/Caffeine	Log(URXAMU)/Caffeine	Log (Theophylline)/Caffeine	Log (Paraxanthine)/Caffeine	Log (Theobromine)/Caffeine	Log (1-methyluric acid^**^)/Caffeine	Log(URXAMU)/Caffeine	Log (Theophylline)/Caffeine	Log (Paraxanthine)/Caffeine	Log (Theobromine)/Caffeine	Log (1-methyluric acid)/Caffeine	Log(URXAMU)/Caffeine
		Beta (P)	Beta (P)	Beta (P)	Beta (P)	Beta (P)	Beta (P)	Beta (P)	Beta (P)	Beta (P)	Beta (P)	Beta (P)	Beta (P)	Beta (P)	Beta (P)	Beta (P)
**Cotinine (ng/mL)**																
Q1	≤0.015	Ref	Ref	Ref	Ref	Ref	Ref	Ref	Ref	Ref	Ref	Ref	Ref	Ref	Ref	Ref
Q2	0.015–0.044	−0.04 (0.66)	0.06 (0.61)	0.002 (0.99)	0.27 (0.17)	0.31 (0.06)	−0.13 (0.58)	−0.05 (0.84)	−0.11 (0.61)	0.35 (0.30)	0.41 (0.13)	0.99 (0.22)	0.37 (0.68)	−0.53 (0.48)	1.39 (0.23)	2.50 (0.05)
Q3	0.044−0.988	0.16 (0.18)	0.25 (0.053)	−0.17 (0.31)	0.61 (0.02)	0.59 (0.03)	−0.12 (0.55)	0.004 (0.98)	−0.28 (0.25)	0.03 (0.93)	0.06 (0.82)	0.75 (0.37)	0.25 (0.77)	1.26 (0.28)	2.62 (0.05)	3.13 (0.01)
Q4	>0.988	−0.32 (0.17)	−0.22 (0.34)	−0.72 (0.04)	0.28 (0.39)	0.42 (0.19)	−0.0002 (1.00)	0.15 (0.56)	−0.34 (0.32)	−0.26 (0.20)	−0.3 (0.13)	−0.68 (0.27)	−1.04 (0.12)	−0.60 (0.43)	1.83 (0.03)	2.26 (0.01)
**P for trend**		0.86	0.54	0.03	0.05	0.03	0.92	0.54	0.28	0.22	0.16	0.04	0.03	0.02	0.51	0.47
**Total Smoking Intensity (cigs)**																
Q1	Former: ≤15,059; Current: ≤26,068						Ref	Ref	Ref	Ref	Ref	Ref	Ref	Ref	Ref	Ref
Q2	Former: 15,059−60,878; Current: 26,068−72,238						−0.03 (0.87)	−0.07 (0.66)	0.07 (0.78)	0.48 (0.28)	0.17 (0.68)	−0.33 (0.25)	−0.42 (0.18)	−0.48 (0.16)	−0.35 (0.24)	−0.37 (0.21)
Q3	Former: 60,878−175,09; Former: 72,238−212,420						0.36 (0.06)	0.35 (0.07)	0.36 (0.26)	0.46 (0.22)	0.46 (0.22)	−0.6 (0.02)	−0.6 (0.03)	−0.77 (0.02)	−0.67 (0.03)	−0.45 (0.12)
Q4	Current: >175,091; Current: >212,420						0.35 (0.13)	0.32 (0.08)	0.13 (0.66)	0.17 (0.49)	0.06 (0.81)	−0.92 (0.01)	−1.01 (0.01)	−0.87 (0.03)	−0.8 (0.04)	−0.68 (0.10)
**P for trend**							0.09	0.048	0.52	0.49	0.59	0.006	0.004	0.03	0.04	0.13

We used the day 1 caffeine intake to calculate caffeine metabolite indices. The covariates adjusted in multivariable models included age, gender, ethnicity, body mass index, physical activity, diabetes, hypertension status, liver condition, glomerular filtration rate, and fasting status. Serum cotinine is used to assess secondhand smoke. Total smoking intensity is calculated separately for former and current smokers.

## Data Availability

The data presented in this study is openly available online at [https://wwwn.cdc.gov/Nchs/Nhanes/2009-2010/ (21 February 2021)].

## Supplementary Materials

The following are available online at https://www.mdpi.com/1660-4601/18/5/2225/s1. Table S1: Serum liver enzymes and caffeine metabolite indices (caffeine intake was calculated from the 24-h recall on day 2).
