# Supplementary material for: Tobacco Smoke and CYP1A2 Activity in a US Population with Normal Liver Enzyme Levels
Source: Int J Environ Res Public Health. 2021 Feb 24;18(5):2225. doi: 10.3390/ijerph18052225 (PMC7956356; doi:10.3390/ijerph18052225)
Supplement: Supplementary file 1 [file ijerph-18-02225-s001.pdf]

Table S1. Serum liver enzymes and caffeine metabolite indices (caffeine intakes was calculated from the 24-hour recall on day 2).

		Never Smoker					Past Smoker					Current Smoker				
		Log (Theophylline) /Caffeine	Log (Paraxanthine)/ Caffeine	Log (Theobromine)/ Caffeine	Log (1-methyluric acid*)/Caffeine	Log(URXAMU) /Caffeine	Log (Theophylline) /Caffeine	Log (Paraxanthine)/Caffeine	Log (Theobromine)/Caffeine	Log (1-methyluric acid)/Caffeine	Log(URXAMU) /Caffeine	Log (Theophylline) /Caffeine	Log (Paraxanthine)/Caffeine	Log (Theobromine)/Caffeine	Log (1-methyluric acid*)/Caffeine	Log(URXAMU) /Caffeine
		Beta (P)	Beta (P)	Beta (P)	Beta (P)	Beta (P)	Beta (P)	Beta (P)	Beta (P)	Beta (P)	Beta (P)	Beta (P)	Beta (P)	Beta (P)	Beta (P)	Beta (P)
**Alkaline Phosphatase (U/L)**																
Abnormal	<30	0.33 (0.64)	0.47 (0.47)	-0.14 (0.87)	1.34 (0.09)	1.28 (0.06)	0.23 (0.75)	-0.08 (0.91)	-0.42 (0.66)	1.70 (0.06)	0.94 (0.30)	-1.82 (0.01)	-1.52 (0.03)	-1.52 (0.11)	-0.15 (0.88)	-0.79 (0.35)
Q1	30 to <55.29	Ref	Ref	Ref	Ref	Ref	Ref	Ref	Ref	Ref	Ref	Ref	Ref	Ref	Ref	Ref
Q2	55.29 to < 68.64	-0.19 (0.19)	-0.14 (0.40)	0.23 (0.22)	0.33 (0.32)	0.23 (0.53)	0.20 (0.46)	0.27 (0.36)	0.41 (0.21)	0.49 (0.04)	0.59 (0.03)	-0.53 (0.002)	-0.42 (0.03)	-0.47 (0.06)	-0.63 (0.15)	-0.7 (0.15)
Q3	68.64 to <86.05	-0.33 (0.16)	-0.30 (0.19)	0.04 (0.89)	-0.10 (0.72)	-0.05 (0.89)	0.21 (0.46)	0.22 (0.47)	0.30 (0.17)	1.19 (0.0001)	1.11 (0.0001)	-0.13 (0.43)	-0.03 (0.89)	-0.19 (0.54)	0.03 (0.93)	-0.01 (0.97)
Q4	≥ 86.05	-0.10 (0.54)	-0.05 (0.77)	0.16 (0.25)	-0.01 (0.99)	0.06 (0.88)	-0.12 (0.6)	-0.11 (0.67)	-0.15 (0.59)	0.44 (0.16)	0.34 (0.30)	0.26 (0.25)	0.52 (0.08)	0.45 (0.32)	-0.06 (0.89)	0.04 (0.93)
**P for trend**		0.09	0.08	0.70	0.47	0.58	0.50	0.60	0.58	0.004	0.007	0.07	0.02	0.09	0.21	0.21
**Aspartate Aminotransferase (U/L)**																
Q1	<19.46	Ref	Ref	Ref	Ref	Ref	Ref	Ref	Ref	Ref	Ref	Ref	Ref	Ref	Ref	Ref
Q2	19.46 to <22.85	-0.14 (0.45)	-0.09 (0.65)	-0.38 (0.13)	0.07 (0.73)	-0.03 (0.89)	-0.26 (0.32)	-0.46 (0.10)	-0.75 (0.04)	-0.39 (0.16)	-0.47 (0.07)	0.20 (0.36)	0.15 (0.52)	0.01 (0.98)	-0.46 (0.29)	-0.45 (0.29)
Q3	22.8 to <27.43	0.22 (0.09)	0.25 (0.07)	-0.16 (0.47)	-0.09 (0.56)	-0.12 (0.54)	-0.08 (0.67)	-0.14 (0.35)	-0.48 (0.08)	-0.61 (0.01)	-0.71 (0.03)	0.53 (0.03)	0.43 (0.12)	0.42 (0.27)	0.33 (0.42)	0.26 (0.53)
Q4	27.43 to <40	0.19 (0.37)	0.25 (0.29)	-0.10 (0.74)	-0.10 (0.54)	-0.17 (0.34)	-0.2 (0.33)	-0.23 (0.21)	-0.25 (0.47)	-0.27 (0.30)	-0.23 (0.45)	0.55 (0.003)	0.52 (0.02)	0.35 (0.16)	0.27 (0.51)	0.37 (0.36)
Abnormal	≥ 40	0.58 (0.07)	0.35 (0.31)	0.06 (0.89)	-0.34 (0.43)	-0.26 (0.59)	-0.11 (0.84)	-0.42 (0.46)	-1.22 (0.06)	-0.48 (0.55)	-0.58 (0.50)	-0.0005 (0.999)	-0.22 (0.61)	-0.3 (0.53)	0.01 (0.99)	-0.12 (0.83)
**P for trend**		0.44	0.27	0.006	0.002	0.001	0.51	0.50	0.69	0.43	0.55	0.30	0.33	0.47	0.71	0.76

		Never Smoker					Past Smoker					Current Smoker				
		Log (Theophylline) /Caffeine	Log (Paraxanthine)/ Caffeine	Log (Theobromine)/ Caffeine	Log (1-methyluric acid**)/Caffeine	Log(URXAMU) /Caffeine	Log (Theophylline) /Caffeine	Log (Paraxanthine)/Caffeine	Log (Theobromine)/Caffeine	Log (1-methyluric acid)/Caffeine	Log(URXAMU) /Caffeine	Log (Theophylline) /Caffeine	Log (Paraxanthine)/Caffeine	Log (Theobromine)/Caffeine	Log (1-methyluric acid**)/Caffeine	Log(URXAMU) /Caffeine
		Beta (P)	Beta (P)	Beta (P)	Beta (P)	Beta (P)	Beta (P)	Beta (P)	Beta (P)	Beta (P)	Beta (P)	Beta (P)	Beta (P)	Beta (P)	Beta (P)	Beta (P)
**Alanine Aminotransferase (U/L)**																
Q1	<15.34	Ref	Ref	Ref	Ref	Ref	Ref	Ref	Ref	Ref	Ref	Ref	Ref	Ref	Ref	Ref
Q2	15.34 to <19.73	0.10 (0.63)	0.13 (0.48)	0.07 (0.79)	0.07 (0.62)	0.15 (0.29)	-0.57 (0.09)	-0.37 (0.21)	-0.24 (0.46)	-0.13 (0.64)	0.05 (0.86)	0.35 (0.02)	0.27 (0.14)	0.35 (0.20)	-0.46 (0.27)	-0.61 (0.14)
Q3	19.73 to <27.09	0.15 (0.46)	0.21 (0.35)	0.09 (0.73)	-0.17 (0.40)	-0.14 (0.52)	-0.40 (0.11)	-0.26 (0.31)	-0.35 (0.42)	-0.71 (0.01)	-0.57 (0.07)	0.32 (0.16)	0.17 (0.38)	0.35 (0.32)	0.57 (0.11)	0.53 (0.12)
Q4	27.09 to <40	0.33 (0.14)	0.34 (0.13)	0.16 (0.38)	0.27 (0.36)	0.30 (0.27)	-0.66 (0.049)	-0.46 (0.14)	-0.51 (0.26)	-0.12 (0.68)	0.22 (0.54)	0.96 (0.01)	0.85 (0.01)	1.40 (0.03)	1.01 (0.07)	1.12 (0.06)
Abnormal	≥ 40	0.12 (0.61)	0.23 (0.32)	0.21 (0.52)	0.29 (0.31)	0.23 (0.40)	-0.75 (0.11)	-0.67 (0.15)	-0.37 (0.55)	-0.56 (0.35)	-0.48 (0.45)	0.40 (0.25)	0.44 (0.21)	0.74 (0.18)	0.48 (0.18)	0.56 (0.11)
**P for trend**		0.43	0.51	0.18	0.27	0.18	0.25	0.40	0.57	0.89	0.59	0.45	0.55	0.26	0.40	0.24
**Aspartate / Alanine Aminotransferase**																
Q1	<0.92	Ref	Ref	Ref	Ref	Ref	Ref	Ref	Ref	Ref	Ref	Ref	Ref	Ref	Ref	Ref
Q2	0.92 to <1.14	0.10 (0.56)	0.06 (0.72)	-0.08 (0.68)	-0.07 (0.75)	-0.11 (0.64)	0.31 (0.06)	0.35 (0.07)	-0.08 (0.68)	-0.26 (0.47)	-0.17 (0.61)	-0.41 (0.08)	-0.41 (0.04)	-0.93 (0.02)	-0.34 (0.22)	-0.51 (0.16)
Q3	1.14 to <1.37	0.11 (0.61)	0.02 (0.92)	0.27 (0.36)	-0.30 (0.24)	-0.40 (0.10)	0.35 (0.12)	0.42 (0.12)	0.27 (0.36)	-0.12 (0.62)	-0.24 (0.28)	0.05 (0.87)	0.07 (0.81)	-0.37 (0.31)	-0.76 (0.01)	-1.03 (0.01)
Q4	≥ 1.37	0.29 (0.24)	0.24 (0.31)	0.05 (0.82)	-0.11 (0.70)	-0.11 (0.72)	0.60 (0.02)	0.52 (0.03)	0.05 (0.82)	0.05 (0.91)	0.04 (0.94)	0.09 (0.72)	0.02 (0.95)	-0.75 (0.09)	-0.74 (0.20)	-1.07 (0.07)
**P for trend**		0.10	0.28	0.52	0.66	0.28	0.40	0.43	0.11	0.13	0.07	0.03	0.07	0.09	0.13	0.07
**Bilirubin (U/L)**																
Q1	<0.52	Ref	Ref	Ref	Ref	Ref	Ref	Ref	Ref	Ref	Ref	Ref	Ref	Ref	Ref	Ref
Q2	0.52 to <0.650	-0.25 (0.32)	-0.25 (0.30)	-0.44 (0.12)	0.14 (0.67)	0.21 (0.49)	0.17 (0.67)	0.05 (0.88)	0.22 (0.60)	0.69 (0.11)	0.76 (0.13)	0.11 (0.70)	0.17 (0.62)	0.49 (0.27)	-0.29 (0.48)	0.04 (0.94)
Q3	0.650 to <0.82	-0.24 (0.21)	-0.25 (0.24)	-0.63 (0.01)	-0.43 (0.19)	-0.36 (0.24)	0.06 (0.84)	0.07 (0.84)	-0.002 (0.995)	0.19 (0.60)	0.04 (0.92)	-0.05 (0.78)	-0.14 (0.46)	-0.24 (0.50)	-1.35 (0.0001)	-0.91 (0.01)
Q4	0.82 to <1	-0.07 (0.77)	0.04 (0.89)	-0.38 (0.11)	-0.07 (0.81)	0.03 (0.93)	0.58 (0.08)	0.50 (0.17)	0.25 (0.36)	0.96 (0.03)	0.88 (0.06)	0.28 (0.35)	0.22 (0.52)	0.04 (0.93)	-1.06 (0.06)	-0.94 (0.14)

		Never Smoker					Past Smoker					Current Smoker				
		Log (Theophylline) /Caffeine	Log (Paraxanthine)/ Caffeine	Log (Theobromine)/ Caffeine	Log (1-methyluric acid*)/Caffeine	Log(URXAMU) /Caffeine	Log (Theophylline) /Caffeine	Log (Paraxanthine)/Caffeine	Log (Theobromine)/Caffeine	Log (1-methyluric acid)/Caffeine	Log(URXAMU) /Caffeine	Log (Theophylline) /Caffeine	Log (Paraxanthine)/Caffeine	Log (Theobromine)/Caffeine	Log (1-methyluric acid*)/Caffeine	Log(URXAMU) /Caffeine
		Beta (P)	Beta (P)	Beta (P)	Beta (P)	Beta (P)	Beta (P)	Beta (P)	Beta (P)	Beta (P)	Beta (P)	Beta (P)	Beta (P)	Beta (P)	Beta (P)	Beta (P)
Abnormal	≥ 1	-0.46 (0.06)	-0.44 (0.07)	-0.55 (0.13)	-0.36 (0.24)	-0.41 (0.23)	0.33 (0.28)	0.34 (0.26)	0.03 (0.92)	0.72 (0.16)	0.82 (0.17)	0.15 (0.63)	-0.15 (0.62)	-0.52 (0.17)	-0.76 (0.14)	-0.42 (0.46)
**P for trend**		0.31	0.51	0.40	0.57	0.57	0.37	0.68	0.23	0.78	0.74	0.90	0.24	0.02	0.001	0.01
**Gamma-glutamyl transpeptidase (U/L)**																
Q1	Female: <12.55; Male: <12.55	Ref	Ref	Ref	Ref	Ref	Ref	Ref	Ref	Ref	Ref	Ref	Ref	Ref	Ref	Ref
Q2	12.55 to <17.94	-0.03 (0.88)	-0.05 (0.78)	-0.16 (0.52)	-0.37 (0.24)	-0.27 (0.35)	-0.16 (0.52)	-0.11 (0.62)	0.42 (0.29)	-0.23 (0.53)	0.05 (0.90)	-0.16 (0.45)	-0.27 (0.32)	0.34 (0.30)	0.03 (0.92)	0.15 (0.67)
Q3	17.94 to <27.71	-0.15 (0.44)	-0.21 (0.27)	-0.34 (0.23)	-0.33 (0.23)	-0.25 (0.33)	0.15 (0.53)	0.11 (0.69)	0.78 (0.07)	0.18 (0.37)	0.48 (0.12)	0.25 (0.20)	0.06 (0.79)	0.45 (0.06)	0.43 (0.18)	0.51 (0.09)
Q4	Female: 27.71-40; Male: 27.71-50	0.20 (0.27)	0.11 (0.50)	-0.05 (0.81)	0.08 (0.69)	0.22 (0.33)	-0.31 (0.26)	-0.26 (0.33)	0.31 (0.29)	0.13 (0.56)	0.33 (0.24)	-0.17 (0.52)	-0.27 (0.36)	-0.01 (0.97)	0.42 (0.34)	0.42 (0.31)
Abnormal	Female: ≥ 40; Male: ≥50	-0.06 (0.79)	-0.13 (0.6)	-0.49 (0.13)	-0.45 (0.12)	-0.31 (0.34)	0.42 (0.20)	0.24 (0.51)	0.73 (0.11)	0.10 (0.82)	0.72 (0.15)	0.11 (0.71)	-0.32 (0.37)	-0.49 (0.29)	-0.11 (0.83)	-0.23 (0.64)
**P for trend**		0.04	0.17	0.97	0.12	0.03	0.03	0.08	0.02	0.29	0.09	0.98	0.23	0.12	0.44	0.54

We used the day 2 caffeine intake to calculate caffeine metabolite indices. The covariates adjusted in multivariable models included age, gender, ethnicity, body mass index, physical activity, diabetes, hypertension status, liver condition, glomerular filtration rate, fasting status, serum cotinine, and total smoking intensity. Total smoking intensity is calculated separately for former and current smokers. Abbreviations: URXAMU: 5-acetylamino-6-amino-3-methyluracil.
